# Increase in the Intracellular Bulk Water Content in the Early Phase of Cell Death of Keratinocytes, Corneoptosis, as Revealed by 65 GHz Near-Field CMOS Dielectric Sensor

**DOI:** 10.3390/molecules27092886

**Published:** 2022-04-30

**Authors:** Keiichiro Shiraga, Yuichi Ogawa, Shojiro Kikuchi, Masayuki Amagai, Takeshi Matsui

**Affiliations:** 1 Graduate School of Agriculture, Kyoto University, Kyoto 606-8502, Japan; ogawa.yuichi.4u@kyoto-u.ac.jp; 2 RIKEN Center for Integrative Medical Sciences, Yokohama 230-0045, Japan; amagai@keio.jp (M.A.); matsuitks@stf.teu.ac.jp (T.M.); 3 PRESTO, Japan Science and Technology Agency, Kawaguchi 332-0012, Japan; 4 Institute for Advanced Medical Sciences, Hyogo College of Medicine, Nishinomiya 663-8501, Japan; skikuchi@hyo-med.ac.jp; 5 Department of Dermatology, Keio University School of Medicine, Tokyo 160-8582, Japan; 6 School of Bioscience and Biotechnology, Tokyo University of Technology, Tokyo 192-0982, Japan

**Keywords:** bulk water content, near-field CMOS dielectric sensor, fluorescence imaging, corneoptosis, SG1 cell

## Abstract

While bulk water and hydration water coexist in cells to support the expression of biological macromolecules, how the dynamics of water molecules, which have long been only a minor role in molecular biology research, relate to changes in cellular states such as cell death has hardly been explored so far due to the lack of evaluation techniques. In this study, we developed a high-precision measurement system that can discriminate bulk water content changes of ±0.02% (0.2 mg/cm^3^) with single-cell-level spatial resolution based on a near-field CMOS dielectric sensor operating at 65 GHz. We applied this system to evaluate the temporal changes in the bulk water content during the cell death process of keratinocytes, called corneoptosis, using isolated SG1 (first layer of stratum granulosum) cells in vitro. A significant irreversible increase in the bulk water content was observed approximately 1 h before membrane disruption during corneoptosis, which starts with cytoplasmic high Ca^2+^ signal. These findings suggest that the calcium flux may have a role in triggering the increase in the bulk water content in SG1 cells. Thus, our near-field CMOS dielectric sensor provides a valuable tool to dissect the involvement of water molecules in the various events that occur in the cell.

## 1. Introduction

Since the birth of primitive life in the ocean about 3.5 billion years ago, life has evolved on the basis of water. It is known that chemical reactions in living organisms would not be possible without water, which forms the medium that accounts for most of the weight of mammalian cells [1]. One of the most important roles of water molecules is the hydration of biological macromolecules, as their conformation is stabilized by the presence of surrounding hydration water [2]. Hence, the hydration environment in the cell is considered to be a mirror of the cell activity [3,4,5], and some theoretical studies have predicted that most of the water in a cell crowded with many biomolecules exists as dynamically restrained hydration water [6,7]. However, in the past 15 years, sporadic studies have experimentally evaluated water dynamics in cells, revealing that the majority of intracellular water molecules exhibit bulk-like orientation and translational motions, with only a fraction of hydration water [8,9,10,11,12,13,14]. This result suggests that both bulk water and hydration water interacting with biomolecules are involved in some way in intracellular activities, and therefore, the equilibrium between bulk and hydration water may play an important biological role in cellular functions.

Research to assess the amount of bulk and hydration water in cells originated in nuclear magnetic relaxation and neutron scattering experiments in the 2000s [8,9,10,11]. However, these techniques require deuterium substitution or tissue freezing to highlight intracellular water dynamics, and have not been able to observe intracellular water in its true physiological state. Later, using terahertz spectroscopy [15,16,17], it was shown that about a quarter of intracellular water in cultured human-derived cells under physiological conditions exists as hydration water, but its uncertainty (more than ±5%) would not be small enough to observe slight changes in the hydration state accompanied by changes in cell conditions [17]. In addition, the wavelength of terahertz waves (300 μm at 1 THz) is sufficiently large for the size of a cell and so it is difficult to obtain spatial resolution at the level of a single cell with far-field spectroscopy, and thus, the water dynamics that can be obtained are limited to the spatial average of a cell monolayer. Therefore, in order to clarify how intracellular water is involved in cellular functions, an improved technique to evaluate the bulk or hydration water content in cells with high precision at single-cell-level spatial resolution is required.

In recent years, we have found that the complex dielectric constant, $\tilde{\varepsilon }=\varepsilon^{\prime }-i\varepsilon^{\prime \prime }$, in the millimeter-wave region (30–300 GHz) can be used to quantitatively evaluate the bulk water content without interference from macromolecules and hydration water (including confined water), because the contribution of dipolar relaxation of bulk water is far more dominant in this region [18,19,20,21]. We developed a dielectric sensor with embedded LC resonators oscillating at ~65 GHz, whose resonance conditions change to reflect the complex dielectric constant of the sample, and analyzed the change in the bulk water content based on the change in resonance frequency, thereby reducing the uncertainty of the evaluated bulk water content by an order of magnitude compared to conventional spectroscopic measurements [20]. The sensor consists of a two-dimensional array of resonators that sense the dielectric responses of the near-field, and the information of all elements can be read out in a CMOS circuit, so that the complex dielectric constant in the 65 GHz region (wavelength of 4.6 mm) can be recognized with a spatial resolution of about 50 μm at video rate [22]. Therefore, this new dielectric sensor can be expected to be an elemental technology for highly precise real-time evaluation of the bulk water content changes occurring in a single cell, which will help elucidate the role of intracellular water.

In this study, we aim to evaluate the relationship between changes in cell state and in the intracellular bulk water content using the near-field CMOS dielectric sensor operating at 65 GHz, using cell death as an example, which involves enormous and irreversible changes in cell physiology. Since changes in the position and morphology of cells may have a significant effect on the measurement results when using near-field sensors, we focus on corneoptosis, which is a process by which non-migratory SG1 cells (keratinocytes located in the superficial layer of stratum granulosum of the skin epidermis) die under high [Ca^2+^] and acidic conditions to transform into nonviable anuclear corneocytes that perform barrier functions in the epidermis [23,24]. In this study, we develop an experimental system that can characterize the bulk water content with ultimate precision using the 65 GHz near-field CMOS dielectric sensor, and present changes in the bulk water content during corneoptosis of SG1 cells isolated from mouse epidermis, in parallel with time-lapse fluorescence imaging.

## 2. Materials and Methods

### 2.1. Near-Field CMOS Dielectric Sensor Operating at 65 GHz

**Figure 1:**
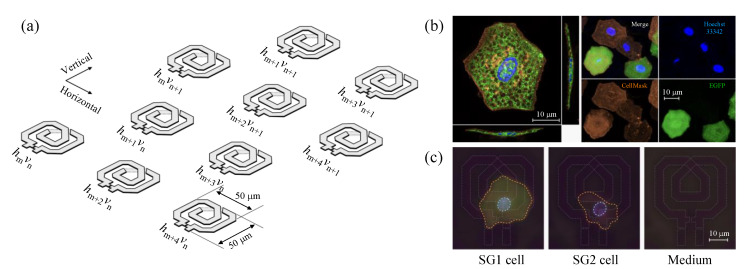
(**a**) Illustration of the LC resonator array. Each resonator element is numbered by *h*_m_*v*_n_ (m-th in horizontal and n-th in vertical direction; 0 ≦ m ≦ 62, 0 ≦ n ≦ 24), and even- and odd-numbered columns in the same row are vertically shifted in a zigzag arrangement. (**b**) Left: Confocal fluorescence images of isolated SG1 cells (EGFP; green) stained with Hoechst 33342 (blue) and CellMask (orange). Note that the scale of the cross-section planes is compressed to 50% compared to that of the top view. Right: Tiled fluorescent labeling of SG1/SG2 cells stained with Hoechst 33342 and CellMask. EGFP expression allows SG1 cells (EGFP-positive) to be fluorescently distinguished from SG2 cells (EGFP-negative). (**c**) Upright fluorescence microscopy images of measured SG1 (EGFP-positive; green) and SG2 (EGFP-negative) cells that meet the “single cell on a single element” condition at t= 0 h. Both cells are stained with Hoechst 33342 (blue), CellMask (orange), and DRAQ7 (magenta). The contours of the resonator structures, cells, and nuclei recognized by these fluorescent dyes are manually traced with white, orange, and cyan lines.

The CMOS dielectric sensor used in this study was a custom-made product, manufactured by Sharp Corporation, with a measurement area of 3 × 4 mm on a 50 × 65 mm sensor chip, in which LC resonators operating at around 65 GHz are arrayed in 24 rows, with even- and odd-numbered rows vertically shifted by forming a zigzag arrangement within each row, as illustrated in Figure 1a [20]. Each LC resonator is coupled to a voltage-controlled oscillator (VCO) and embedded in a frequency synthesizer loop, and its oscillation frequency is detected by a 32-bit frequency counter after down-conversion to ~100 MHz output frequency by divide-by-3 injection-locked frequency divider and divide-by-200 divider [22]. This LC resonator, defined by the capacitance $C_{0}$ and inductance $L_{0}$, is implemented in a top metal layer, on which a passivation layer (capacitance: $C_{1}$) of several μm thickness is formed. When the measurement sample with $C_{2}-iG_{2}\propto \varepsilon_{2}^{\prime }-i\varepsilon_{2}^{\prime \prime }$ is applied on it, the resonant frequency f of this LC resonator is given by [20,22]:

(1) $$f={\left[2\pi \sqrt{L_{0}\left\{C_{0}+C_{1}\frac{C_{1}C_{2}+C_{2}{}^{2}+G_{2}{}^{2}}{{\left(C_{1}+C_{2}\right)}^{2}+G_{2}{}^{2}}\right\}}\right]}^{-1}$$

Equation (1) indicates that each LC resonator acts as a sensing element that measures the complex dielectric constant of the sample through the resonant frequency f. The $C_{0}$ and $L_{0}$ of the resonators are strictly locked in a self-sustained manner according to the VCO, and even with a gating time of 200 μs, the variation in the resonant frequency is limited within ±0.17 MHz (under ideal circumstances), which is extremely small compared to the center frequency (~65 GHz) [22]. Given that the impact of dipolar relaxation on the complex dielectric constant ($\varepsilon_{2}^{\prime }$ and $\varepsilon_{2}^{\prime \prime }$) in the 65 GHz region is sufficiently larger for bulk water than for macromolecules or hydration water [18,19,20,21], each resonator element serves as a highly sensitive dielectric sensor that can characterize the bulk water content by distinguishing small changes in the complex dielectric constant in the 65 GHz region, and the two-dimensional spatial distribution of the bulk water content can be also evaluated in real time by reading out a total of 1488 arrayed elements in the CMOS circuit. However, the detection sensitivity of this dielectric sensor is not spatially uniform; the dielectric properties of the sample near the gap of the LC resonator are most sensitive to the resonance frequency in the planar direction, and the detection sensitivity decays exponentially with depth [22]. As a result, each LC resonator works as a near-field sensor reflecting the complex dielectric constant at around 65 GHz in a region limited to ~50 μm in diameter and ~10 μm depth, which is determined by the resonator structure and the thickness of the passivation layer, thus enabling the precise evaluation of the bulk water content with single-cell-level spatial resolution as long as the cell is located in the sensitive area of the LC resonator.

### 2.2. Experimental Setup of Fluorescence Imaging

To assess the morphology of the cells by acquiring the fluorescence images in parallel with the dielectric sensor measurements, we set up a custom-made Tokai Hit imaging chamber on the manual translation stage of a Nikon upright microscope (Eclipse Ni-U). Inside the chamber was kept at 37 °C with humidified air containing 5% CO_2_ supplied from a controller STGX (Tokai Hit, Fujimiya, Japan), and the dielectric sensor chip was mounted on the substrate in the chamber. A stainless steel well with a diameter of 16 mm and a depth of 5 mm is fixed around the measurement area of the dielectric sensor as a liquid reservoir. A 20× water immersion lens (CFI Fluor 20× W, Nikon, Tokyo, Japan) is approached through an opening in the chamber canopy until it is immersed in the liquid filling the well, allowing acquisition of the fluorescence image of the dielectric sensor chip surface in the upright position.

### 2.3. Preparation of SG1/SG2 Cell Suspension

By injecting exfoliative toxin-A into the dorsal skin of 4-month-old EGFP^SG1^ knock-in mice (male), epidermal sheets (approximately 5 × 15 mm) containing SG2 cells (keratinocytes in the 2nd layer of stratum granulosum) and SC cells (keratinized dead cells; corneocytes) as well as SG1 cells were detached [23], and all cells were separated from the sheets in trypsin solution containing Hoechst 33342 and CellMask Orange Plasma (Thermo Fisher Scientific Inc., Waltham, MA, USA) at final concentrations of 10 μg/mL and 15 μg/mL, respectively. After that, the extracellular [Ca^2+^] was reduced to less than ca. 1 nM by adding 1 mM ethylene glycol tetraacetic acid, and then SG1/SG2 cell suspensions were prepared by adding MCDB 153 medium at pH 7.2 containing 3 μM DRAQ7 (Biostatus Ltd., Shepshed, UK) after centrifuge. As shown in the confocal images in Figure 1b, SG1 cells isolated by this procedure express EGFP in the entire cytoplasm except for numerous intracellular granules, whereas SG2 cells are EGFP-negative. In the skin epidermis, SG1 cells are known to have a flattened Kelvin’s tetrakaidecahedron-like shape [25], but during the isolation process, the expansion rate differs between the apical and basolateral side of the cells, resulting in a polygonal saucer-like morphology. While SG1 cells can be cultured in a viable state, SG2 cells are dead in the final cell suspension, presumably because the cell membrane is directly damaged for a longer period of time than SG1 cells during trypsin treatment.

### 2.4. Simultaneous Evaluation of SG1 Cell Death by Dielectric Sensor and Microscopy

We applied 600 μL of the SG1/SG2 cell suspension onto the measurement area of the dielectric sensor placed at the focal point of the upright microscope and waited for about 10 min until the cells were completely settled. After that, by observing the entire measurement area (3 × 4 mm) while manually moving the translation stage of the microscope, we identified 13 resonator elements that had only one cell on the sensitive area, i.e., elements that satisfied the “single cell on a single element” condition. By discriminating SG1 cells from SG2 based on the EGFP expression, we found that 7 of the 13 cells were SG1 cells and the remaining 6 were SG2 cells. After fixing the manual stage in the field of view where one SG1 cell and one SG2 cell (Figure 1c), each of which fulfilled the “single cell on a single element” condition, could be observed simultaneously, corneoptosis of SG1 cells was induced by gently replacing the medium in the wells with MCDB 153 medium at pH 6.1 supplemented with 1 mM CaCl_2_ and 3 μM DRAQ7 at final concentration. After the entire surface of the sensor was observed within 10 min to ensure that no change in the position of the cells had occurred due to the replacement of the medium, simultaneous data acquisition of the dielectric sensor and upright fluorescence microscope was performed at 10-minute intervals with the initial measurement, t= 0 h, until the 16th hour in total. Since the dielectric sensor scans all 1488 elements in the CMOS circuit in a few seconds, the resonant frequencies of all the elements with seven SG1 cells and six SG2 cells were acquired almost simultaneously. With the fluorescence microscope, on the other hand, the fluorescence of one SG1 and one SG2 cell each was only observed, because it performed fixed-point observation in the same field of view.

## 3. Results and Discussion

### 3.1. Establishment of a High-Precision Evaluation for Resonant Frequency Changes

**Figure 2:**
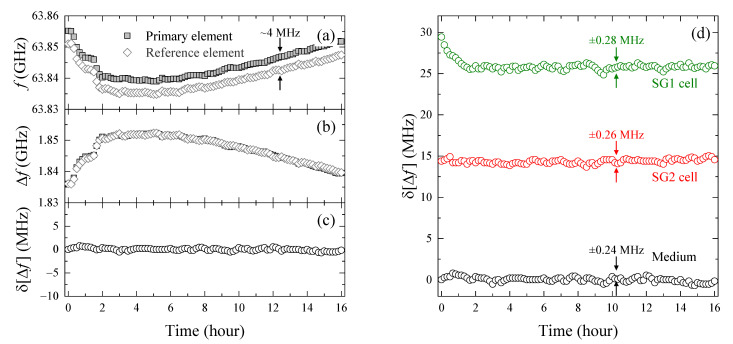
(**a**) Oscillation frequency $f\left(t\right)$ and (**b**) frequency shift $\Delta f\left(t\right)$ of the primary resonator element with the liquid medium on top and its vertically adjacent resonator element (reference element). (**c**) Time-dependent difference frequency shift $\delta \left[\Delta f\left(t\right)\right]$ defined by Equation (3). (**d**) Difference frequency shift $\delta \left[\Delta f\left(t\right)\right]$ for the SG1 cell (green), the dead SG2 cell (red), and liquid medium (gray) observed simultaneously by fluorescence microscopy. Standard deviations over 3–16 h (SG1 cell) and 0–16 h (SG2 cell and medium) are given for reference.

Figure 2a shows the time-dependent change in the resonant frequency f of a randomly selected element with no cells in the sensitive area (hereafter referred to as the “primary element”), which observes only the dielectric responses of the culture medium. The resonant frequency f fluctuated non-monotonically during the 16 h of continuous measurement, with a maximum change of 0.0161 GHz (16.1 MHz). However, since stability of the center frequency of the resonator in this dielectric sensor is typically controlled within ±1 MHz in our experimental setup [20], this 16.1 MHz variation is considered to reflect fluctuations in the dielectric properties of the medium rather than fluctuations in the resonator itself, such as $C_{0}$ and $L_{0}$. The dielectric response of bulk water, which makes up most of the medium, has a pronounced temperature dependence in the 65 GHz region [26], and the resonant frequency f of the dielectric sensor downshifts by about 26 MHz for every 1 °C increase in bulk water temperature [20]. Therefore, a change in the resonance frequency of 16.1 MHz corresponds to about 0.6 °C in terms of temperature, but this level of temperature fluctuation is conventionally tolerated in general cell culture, and besides, it is not technically easy to keep the temperature more strictly constant over 10 h. However, since the LC resonators in the dielectric sensor used in this study are arrayed at 50 μm intervals, the temperature between two adjacent resonators is expected to be extremely close, and by calculating their difference, the effect of temperature fluctuation on the resonant frequency can be compensated over a long period of time. In fact, as shown in Figure 2a, the f of the resonator element vertically adjacent to the primary element (“reference element”) shows a quite similar time variation, keeping a constant difference of ~4 MHz regardless of time, which originates from differences in the device constants such as the resonator ($C_{0}$, $L_{0}$) and the passivation layer ($C_{1}$) for each element. Therefore, in order to take into account this effect, the frequency shift Δf at time t for an individual LC resonator is defined as [20,22]:

(2) $$\Delta f\left(t\right)=f_{0}-f\left(t\right)$$

where $f_{0}$ is the resonance frequency when there is no sample on the resonator. Since the frequency shift Δf represents the change in f of the same element with and without the sample, it can effectively cancel the effect of the device constant. The calculated frequency shifts Δf of the primary and the adjacent reference elements in Figure 2b show that they were in good agreement, indicating that the difference in the resonant frequency f by 4 MHz seen in Figure 2a is indeed due to the difference in the device constants ($C_{0}$, $L_{0}$ and $C_{1}$) between the elements, and there is little difference in the dielectric properties (=temperature) of the sample sensed by the two adjacent elements. Here, we define the difference between the frequency shift $\Delta f_{\mathrm{ref}}\left(t\right)$ of the reference element and the frequency shift $\Delta f\left(t\right)$ of the primary element,

(3) $$\delta \left[\Delta f\left(t\right)\right]=\Delta f_{\mathrm{ref}}\left(t\right)-\Delta f\left(t\right)$$

as the difference frequency shift. As shown in Figure 2c, the $\delta \left[\Delta f\left(t\right)\right]$ calculated from Figure 2b was maintained at 0.02 ± 0.24 MHz for 16 h, allowing precise observation of the change in resonance frequency over time. Using Equations (1)–(3), it is calculated that the variation in the resonance frequency of ±0.24 MHz is comparable with changes of 0.002 in the real part ($\varepsilon_{2}^{\prime }$) and 0.004 in the imaginary part ($\varepsilon_{2}^{\prime \prime }$) of the dielectric constant [20], which are far smaller than the dielectric constant of pure water at 65 GHz (14.9 for the real and 23.7 for the imaginary part at 37 °C). Assuming that the dielectric constant change is solely due to the change in the bulk water content, the frequency shift of ±0.24 MHz corresponds to a ±0.02% (i.e., ~0.2 mg/cm^3^ according to the water density of ~1.0 g/cm^3^) change in the bulk water content using the analysis algorithm established in our previous study [20]. Therefore, this technique can identify changes in the bulk water content as a significant difference if it is 0.02% or more.

### 3.2. Evaluation of Intracellular Bulk Water Content Changes during SG1 Cell Death

Next, the element in which the cell is included in the sensitive area is considered to be the primary element, and its adjacent element with no cells is used as the reference. The difference frequency shift $\delta \left[\Delta f\left(t\right)\right]$ in the live SG1 cell (at t= 0 h) and dead SG2 cells measured while observing with the upright fluorescence microscope, and the same result as in Figure 2c, named medium, are shown in Figure 2d. The medium showed almost zero $\delta \left[\Delta f\left(t\right)\right]$ throughout the entire measurement time, while that of the dead SG2 cells averaged about 15 MHz over time, with a similar time variation (±0.26 MHz). Based on Equation (3), this result indicates that $\Delta f\left(t\right)$ is smaller than the frequency shift $\Delta f_{\mathrm{ref}}\left(t\right)$ of the reference element (liquid medium) by ~15 MHz. Given that the complex dielectric constant in the 65 GHz region decreases in both real and imaginary parts as the bulk water content in the sample decreases [20], which results in a decrease in the frequency shift Δf (or an increase in f), the larger time-averaged difference frequency shift $\delta \langle \Delta f\left(t\right)\rangle$ in the SG2 cell than in the medium indicates that the bulk water content in the SG2 cell is lower than in the liquid medium. This result is considered to be natural because water accounts for about 99% of the weight of the MCDB153 medium [27,28], while the water content is relatively low due to the presence of organelles and some of the water is hydrated to biomolecules in the cells [15,16,17]. As the dielectric sensor used in this study has a spatially non-uniform sensitivity distribution [22], at this point, we are not able to determine the absolute value of the bulk water content in the cell due to the presence of extracellular water, but it is possible to observe the time-dependent change in the bulk water content above 0.02%.

Figure 2d shows that the difference frequency shift $\delta \left[\Delta f\left(t\right)\right]$ in the SG1 cell decreases monotonically until the second hour after induction of corneoptosis at t= 0 h and then converges to a constant value at ~26 MHz, in contrast to the constant $\delta \left[\Delta f\left(t\right)\right]$ of the dead SG2 cell and liquid medium during 16 h of the continuous measurement (the larger $\delta \langle \Delta f\left(t\right)\rangle$ than that of the SG2 cell is because the observed SG1 cell occupies a larger part of the sensitive area of the resonator element). By fitting this result with a single exponential function, it was found that $\delta \left[\Delta f\left(t\right)\right]$ of the SG1 cell decreased by 3.64 MHz with a time constant τ= 0.55 h, which means that it takes 0.55 h for $\delta \left[\Delta f\left(t\right)\right]$ to reach 63.2% (=1$-e^{-1}$) of equilibrium. In light of the above, the significant decrease in $\delta \left[\Delta f\left(t\right)\right]$ during 0 ≦t≦ 2 h indicates that the dielectric sensor reflects some phenomenon related to the initial process of corneoptosis by the SG1 cell.

**Figure 3:**
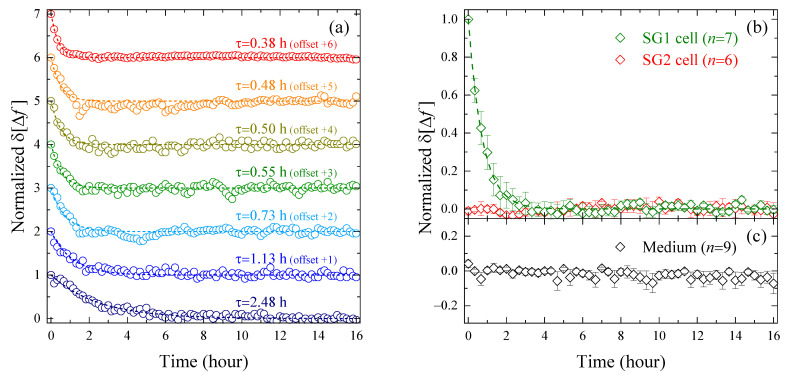
(**a**) Normalized $\delta \left[\Delta f\left(t\right)\right]$ of seven elements with a SG1 cell located in the sensitive area of the resonator (offsetting is applied to avoid data overlap). Each of the experimental results was fitted with the single exponential function with the time constant τ. The data with τ= 0.55 h represent a normalized version of the results shown in Figure 2d. (**b**) Normalized $\delta \left[\Delta f\left(t\right)\right]$ averaged over the examined SG1 cells (n= 7) and SG2 cells (n= 6), and (**c**) liquid medium (n= 9). The error bars represent the standard error.

To confirm whether the monotonic decrease in $\delta \left[\Delta f\left(t\right)\right]$ immediately after the corneoptosis induction is also observed in different cells, the measured $\delta \left[\Delta f\left(t\right)\right]$ of the SG1 cells (n= 7) that met the condition of “single cell on a single element”, as secured by fluorescence microscopy, were collected. In order to evaluate their response to time, ignoring the effect of cell position on the sensitive area of the resonator element, the normalized $\delta \left[\Delta f\left(t\right)\right]$ adjusted to take values between 0 and 1 was derived, and then fitted with the exponential function, as shown in Figure 3a. Considering that SG1 cells isolated from mouse epidermis were used in this study, the time constant τ, which varied from 0.38 to 2.48 h, reflects the heterogeneity among cells. However, the characteristic of the temporal change—$\delta \left[\Delta f\left(t\right)\right]$ monotonically decreasing immediately after cell death induction and then reaching a plateau—was common to all seven SG1 cells. The normalized $\delta \left[\Delta f\left(t\right)\right]$ of the n= 7 average of the SG1 cells, as well as the SG2 cells (n= 6) and liquid medium (n= 9), are summarized in Figure 3b,c. The dead SG2 cells and medium showed almost constant $\delta \left[\Delta f\left(t\right)\right]$ over the 16 h of measurement, but only that of the SG1 cells showed a significant exponential decrease, confirming the reproducibility of the results seen in Figure 2d. Based on this finding, in the following, we will deepen the interpretation of $\delta \left[\Delta f\left(t\right)\right]$ by limiting it to the SG1 cell shown in Figure 2d, and compare it with biological findings obtained by fluorescence labeling.

**Figure 4:**
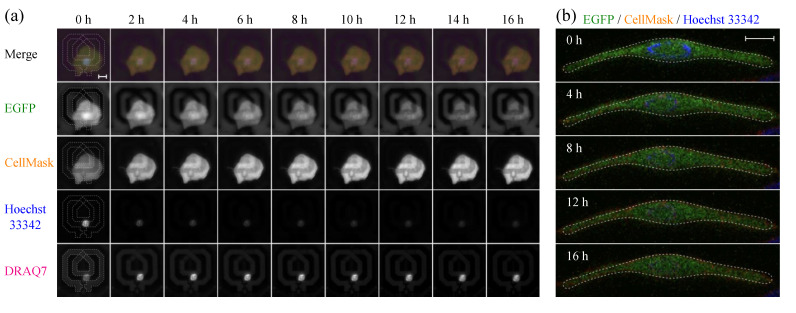
(**a**) Time-lapse fluorescence images of the SG1 cell (EGFP-positive) stained with CellMask (orange), Hoechst 33342 (blue), and DRAQ7 (magenta) under pH 6.1 and 1 mM [Ca^2+^] conditions (scale bar, 10 μm). The resonator structure is manually traced with white dotted line at t= 0 h to show the relative position of the cell (nucleus) and the resonator. (**b**) Cross-sectional images of a SG1 cell isolated from different mice on different days at 4-hour intervals observed with a confocal microscope (scale bar, 10 μm). The cross-sectional outline at 0 h is superimposed to confirm that there are no three-dimensional morphology changes in the cell.

If we assume that the decrease in $\delta \left[\Delta f\left(t\right)\right]$ of the SG1 cell by 3.64 MHz, found in Figure 2d, is solely due to the change in the dielectric properties at around 65 GHz, this result can be interpreted as an increase in the intracellular bulk water content in the early stage of corneoptosis in SG1 cells (according to Equations (1)–(3), $\delta \left[\Delta f\left(t\right)\right]$ decreases as the bulk water content increases, and becomes $\delta \left[\Delta f\left(t\right)\right]=$ 0 MHz when it is the same as the liquid medium). However, each resonator element works as a near-field sensor that is sensitive only to a spatially limited area, and therefore, the dielectric properties of the sample sensed by the resonator change as the relative position of the cell to the resonator element or the thickness of the cell changes. Hence, in order to understand the origin of the $\delta \left[\Delta f\left(t\right)\right]$ observed in Figure 2d, it is essential to observe the changes in cell position and morphology based on microscopy. Figure 4a shows the temporal changes in the upright fluorescence microscope images taken in parallel with the dielectric sensor measurement. The SG1 cells express endogenous EGFP (green) with additional staining by CellMask (red) for the plasma membrane, and membrane-permeable Hoechst 33342 (blue) and membrane-impermeable DRAQ7 (orange) for the nucleus. Figure 4a shows that the isolated SG1 cell do not migrate in parallel or rotate within the measurement time. Furthermore, time-lapse imaging using confocal microscopy of a SG1 cell isolated from different mice on different days revealed that in vitro corneoptosis causes loss of Hoechst 33342 fluorescence but little change in depth and thickness, which is common to all observed cells (n= 7); a cross-sectional image of one of them is shown in Figure 4b. These observations suggest that SG1 cells are not migratory and that their three-dimensional morphology is largely preserved throughout corneoptosis, at least in the in vitro environment. Therefore, we can exclude the possibility that changes in the position or morphology of SG1 cells affect the change in $\delta \left[\Delta f\left(t\right)\right]$, and hence, the decrease in $\delta \left[\Delta f\left(t\right)\right]$ seen in Figure 2d can be attributed to an increased bulk water content in the cell. Calculated in the same manner as above, a decrease in $\delta \left[\Delta f\left(t\right)\right]$ of 3.64 MHz corresponds to an increase in the bulk water content of 0.3% (~3 mg/cm^3^) [20]. Nevertheless, as SG1 cells have a convexly curved shape (Figure 1b) and the liquid medium outside the cells also occupies the sensitive area of the dielectric sensor, the bulk water content in the SG1 cell is considered to increase by more than 0.3% in actuality.

In living cells, cellular stability is accomplished by a number of homeostatic processes, and the cell volume (in other words, the intracellular water content) is maintained within a certain range. However, when cell death is induced, failure of these homeostatic processes results in volume changes specific to the cell death process [29,30]. For example, in apoptosis, monovalent cations, mainly sodium and potassium [31], are released from the cell, and the resulting osmotic shock causes rapid loss of water through aquaporins [32], leading to a tens of percent decrease in cell volume. On the other hand, in corneoptosis of SG1 cells, the nucleus and mitochondria, which are essential for cell survival, are lost, while the keratin network [33] and cornified envelope are developed in the cell [34] and the remaining cell body is taken over by the corneocyte. Thus, the three-dimensional morphology of SG1 cells maintains its mechanical strength even after cell death, and as a result, no discernible volume change is observed in corneoptosis in vitro, unlike other cell deaths. Nevertheless, it is intriguing to note that corneoptosis, as well as other cell deaths such as apoptosis (although the intracellular “bulk” water content was not directly assessed), share the same event of changes in intracellular water content, and it is worth noting what causes the one-step irreversible increase in the bulk water content seen in corneoptosis and what biological significance it has in the cell death process.

### 3.3. Comparison of Changes in Intracellular Water and Cell Membrane Permeability

**Figure 5:**
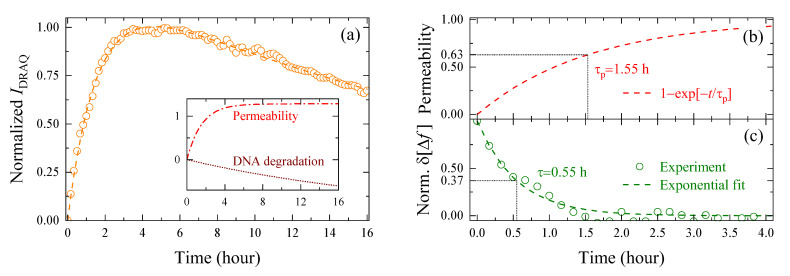
(**a**) Normalized fluorescence intensity of DRAQ7, $I_{\mathrm{DRAQ}}\left(t\right)$, and its best-fitting biexponential functions (dashed lines). The inset shows the time variation of the membrane permeability and DNA degradation components of the biexponential function given by Equation (4). (**b**) Membrane permeability defined by $1-\exp\left[-t/\tau_{p}\right]$, and (**c**) normalized $\delta \left[\Delta f\left(t\right)\right]$ and its exponential fit of the same SG1 cell.

To further understand the relationship between changes in the intracellular bulk water content and biological processes during corneoptosis, we will now quantitatively compare $\delta \left[\Delta f\left(t\right)\right]$ and fluorescence imaging in the same SG1 cell. In this experiment, nuclei of the cells were stained with membrane-permeable Hoechst 33342 and membrane-impermeable DRAQ7, and Figure 4a shows that the former showed strong fluorescence from t= 0 h, while the latter increased its intensity from 0 to 4 h followed by a gradual decrease. In order to interpret this temporal change, the fluorescence intensity of DRAQ7, $I_{\mathrm{DRAQ}}\left(t\right)$, normalized to a value between 0 and 1 is shown in Figure 5a. Consistent with a recent report [23], the rapid increase in $I_{\mathrm{DRAQ}}\left(t\right)$ seen immediately after induction of corneoptosis reflects the binding of DRAQ7 dye to intracellular DNA due to the increase in cell membrane permeability. However, once corneoptosis begins to set in, DNA degradation also progresses due to the activation of deoxyribonuclease, and after the increase in membrane permeability subsides, the effect of DNA degradation becomes apparent as a slow and monotonous decrease in $I_{\mathrm{DRAQ}}\left(t\right)$. In order to make a quantitative comparison with the change in $\delta \left[\Delta f\left(t\right)\right]$ seen in Figure 2d, the normalized $I_{\mathrm{DRAQ}}\left(t\right)$ was fitted with the following biexponential function:

(4) $$I_{\mathrm{DRAQ}}\left(t\right)=\Delta I_{p}\left(1-\exp\left[-t/\tau_{p}\right]\right)+\Delta I_{d}\exp\left[-t/\tau_{d}\right]$$

where the first term on the right-hand side corresponds to the increase in DRAQ7 intensity due to the increased cell membrane permeability, and the second term corresponds to the gradual intensity decrease due to DNA degradation, with ΔI and τ representing the amplitude and time constant of each process, respectively. Based on the best-fitting parameters, the time evolution of cell membrane permeability and DNA degradation is reproduced in the inset of Figure 5a.

The observed membrane permeabilization is not limited to corneoptosis of SG1 cells, but is also observed in other cell deaths [29], and hence, the increased intensity of membrane-impermeable dye is widely used as a probe of cell death accompanied by plasma membrane disruption. Since the permeability of DRAQ7 with a molecular weight of about 400 Da allows smaller water molecules to pass through the cell membrane in and out of the cell, the increase in the bulk water content in the cell may be related to increased membrane permeability. Aiming to evaluate this effect, we compared the membrane permeability, $1-\exp\left[-t/\tau_{p}\right]$, and the normalized frequency shift, $\delta \left[\Delta f\left(t\right)\right]$, of the same SG1 cell in Figure 5b,c. The results show that the time constant of $\delta \left[\Delta f\left(t\right)\right]$ (τ= 0.55 h) is separated from $\tau_{p}=$ 1.55 h by as much as one hour, even though the same cell was observed simultaneously by the dielectric sensor and fluorescence microscope. Matsui et al. recently reported that $\tau_{p}$ was in good agreement with the time constant for the leakage of the fluorescent dye Rhod4-AM out of the cell [23], indicating that $\tau_{p}$ is a direct index of the rate of cell membrane disruption because the time required for DRAQ7 molecules to bind to DNA and show fluorescence after penetrating the cell membrane is sufficiently small compared to $\tau_{p}$. Therefore, the fact that the time constant of $\delta \left[\Delta f\left(t\right)\right]$ was one hour shorter than $\tau_{p}$ suggests that the bulk water content increased in the cell before the permeability of the cell membrane increased. In addition, the fact that $\delta \left[\Delta f\left(t\right)\right]$ reaches a plateau after t= 2 h indicates that the intracellular bulk water content is almost unaffected by membrane permeabilization and DNA degradation.

Previously, it was shown that when isolated SG1 cells were exposed to acidic medium containing 1 mM [Ca^2+^], instantaneous calcium flux into SG1 cells was observed within 5 min, which possibly activates enzymes such as transglutaminase, deoxyribonuclease, and aspartic protease in the cells, resulting in the progression of death into anuclear cells with the cornified envelope [23]. In general, when an enzyme specifically binds to a substrate, a portion of the hydration water immobilized in its active site is displaced [35,36], increasing the number of water molecules that behave in a bulk-like manner [37]. Although it is possible in principle to consider that the increase in the intracellular bulk water content observed in this study is due to such a release of hydration water, the bulk water content increased by enzyme-substrate binding cannot be more than 0.1% (~1 mg/cm^3^) at most since the number of hydration molecules occupying the active site of the enzyme is small, nor can it explain the irreversible change in $\delta \left[\Delta f\left(t\right)\right]$. On the other hand, it has been shown that calcium plays an important regulatory role in the function of aquaporins, which are involved in the rapid volume loss (water outflow) that occurs during apoptosis [38,39]. This makes a possible scenario that elevated cytoplasmic [Ca^2+^] activates aquaporins at the same time that corneoptosis is initiated, causing a water influx into the cell. The irreversible increase in the bulk water content may alter a vital entropy sink for biochemical reactions in the cell [40], resulting in changes in protein folding, self-assembly of membranes, and expression of macromolecular functions [2].

## 4. Conclusions

While physicochemical studies have revealed that water is closely associated to the functional expression of biological macromolecules, little is known about the involvement of water in the actual cell crowded with numerous molecules. In this study, we aimed to understand this issue in a paradoxical way by observing temporal changes of the intracellular bulk water content during SG1 cell death (corneoptosis) into anuclear corneocytes. Using a near-field CMOS dielectric sensor capable of measuring the complex dielectric constant in the 65 GHz region with high sensitivity, we developed a measurement system that can observe changes in the bulk water content of ±0.2 mg/cm^3^. We then found that the bulk water content significantly increases by more than 3 mg/cm^3^ in an isolated SG1 cell in the early stage of corneoptosis, which is triggered by an increase in cytoplasmic [Ca^2+^]. Simultaneous observation of the membrane-impermeable DRAQ7 fluorescence intensity revealed that the time constant τ for the increase in the intracellular bulk water content was found to be about one hour shorter than that for the change in DRAQ7 intensity, indicating that changes in water dynamics in SG1 cells precede membrane permeabilization in corneoptosis. Since such temporal changes in the bulk water content were not observed in dead SG2 cells, it is possible that although living SG1 cells have a mechanism to actively maintain low bulk water content, the induction of cell death by corneoptosis may upset the equilibrium of water dynamics maintained in the cell, probably due to cytoplasmic calcium-induced activation of aquaporin, which in turn promotes changes in the state of macromolecules and hence the progression of cell death.

Although it is not possible to speculate further at this time due to the lack of available experimental evidence, if this state-of-the-art near-field CMOS dielectric sensor, which is still capable of acquiring data with high time resolution at video rates, can be further developed to improve measurement precision and spatial resolution, and if it can be integrated with surface modification technologies to reduce cellular migration, it is expected that further studies using a wide range of cells and bacteria will elucidate, in a real-time and non-invasive manner, the involvement of bulk water in various cell activities, not just cell death.

## Data Availability

The data presented in this study are available on request from the corresponding author.

## Sample Availability

Not available.
